# Telehealth to the Rescue During COVID-19: A Convergent Mixed Methods Study Investigating Patients' Perception

**DOI:** 10.3389/fpubh.2021.730647

**Published:** 2021-11-30

**Authors:** Ghadah A. Al-Sharif, Alia A. Almulla, Eman AlMerashi, Reem Alqutami, Mohammad Almoosa, Mona Zakaria Hegazi, Farah Otaki, Samuel B. Ho

**Affiliations:** ^1^College of Medicine, Mohammed Bin Rashid University of Medicine and Health Sciences, Dubai, United Arab Emirates; ^2^Department of Family Medicine, Mediclinic City Hospital, Dubai, United Arab Emirates; ^3^Strategy and Institutional Excellence, Mohammed Bin Rashid University of Medicine and Health Sciences, Dubai, United Arab Emirates; ^4^Department of Medicine, Mediclinic City Hospital, Dubai, United Arab Emirates

**Keywords:** COVID-19 pandemic, healthcare quality, telehealth, teleconsultation, mixed methods, data integration, joint display analysis, value-based health care

## Abstract

**Background:** The onset of the pandemic necessitated abrupt transition to telehealth consultations. Although there is a few tools that gauge the patients' perception about their experiences, none of them are contextualized to an emergency in the Middle East and North Africa region. Accordingly, this study aims at developing and validating a tool to address this gap, and deploying it to assess the patients' perception of telehealth services during COVID-19 in Dubai, United Arab Emirates (UAE).

**Methods:** A convergent mixed methods design was adapted. A random selection of 100 patients from Dubai, UAE were invited to participate. Qualitative and quantitative datasets were collected using a tailor-made survey. The qualitative data, collected through open-ended questions, was analyzed using multi-staged thematic analysis. As for the quantitative data, it captured the patients' extent of satisfaction, and was assessed using SPSS (with a series of descriptive and inferential analyses). The qualitative and quantitative findings were then merged via joint display analysis.

**Results:** Out of the 100 patients that were randomly selected, 94 patients participated in this study. The reliability score of Cronbach's Alpha for the instrument was 98.9%. The percentage of the total average of satisfaction was 80.67%. The Principal Component Analysis showed that 88.1% of the variance can be explained by the instrument (*p* < 0.001). The qualitative data analysis expanded upon the quantitative findings enabling a better understanding of the patients' perception. Three themes, revolving around the quality of the patient telehealth experiences, surfaced: “Factors that worked to the benefit of the patients,” “Factors that the patients were not in favor of,” and “Opportunities for improvements as perceived by the patients.”

**Discussion:** This study introduced a novel patient satisfaction with telehealth consultation survey contextualized to the COVID-19 times in Dubai, UAE. The participants were quite satisfied with the quality of their experience, however they suggested areas for improvement. Regional healthcare decision-makers can leverage the identified advantages and opportunities for improvement of telehealth. This will enable making informed decisions regarding the continuity of telehealth irrespective of how matters unfold in relation to the pandemic. It will also better prepare the healthcare sector for potential resurgence(s) of COVID-19 and/or the occurrence of other similar emergencies.

## Introduction

Due to the Coronavirus disease 2019 (COVID-19) global spread, the World Health Organization (WHO) officially declared COVID-19 as a pandemic. Based on the WHO guidelines, the United Arab Emirates (UAE) authorities enforced physical distancing directives to control the transmission of the virus and flatten the COVID-19 cases' curve (1–5). This significantly affected the delivery of health services, since the people of the UAE, just like any citizen of the world, still needed to be cared for, health wise.

The delivery of healthcare in Dubai is a shared responsibility between the public and private sectors (6). Prior to COVID-19, the UAE healthcare system was structured in a way where patients need to be physically present at the healthcare delivery systems to get their short- or long-term health care, be it via inpatient or outpatient services (7, 8). There are several studies that highlight that most patients that seek healthcare services in the UAE express satisfaction with the quality of care received (9, 10).

Quality health care can be defined in several ways. Yet, there is acknowledgment that quality health services need to be safe, patient-centered, effective and efficient, timely, and accessible and equitable. A lot of frameworks were developed to measure the quality of healthcare, including the ones proposed by the Institute of Medicine (IOM) and WHO (11, 12). Both frameworks have been frequently referred to in holistic, systemic assessments of traditional, face-to-face healthcare services and aided in Quality Assurance and Continuous Quality Improvement (13–15).

An important strategy learned from previous outbreaks is to protect high-risk citizens from exposure while still maintaining their health and wellbeing in check (16). As such, telehealth has been implemented as a novice means of delivering healthcare services in many hospitals across the UAE in response to the increase in health service demands. Telehealth refers to the means of looking after patients through telecommunication (mainly via a video- or an audio-call), and hence patients do not need to be physically present at a clinic or a hospital. With the current advances in technology, telehealth has a promising future, and is shaping and changing the delivery of remote healthcare in both developed and developing countries (17–20).

On the other hand, and despite telehealth carrying many advantages, its limitations are in creating a rapport between the doctor and patient, as well as performing physical examination, and payment and insurance coverage (17). Other restrictions include possible technical difficulties and security breaches that might interfere with the quality of care delivered, access to reliable technology, as well as lack of a private or confidential space for sessions (21). Telehealth also features many legal and regulatory barriers including differences in rules and practice guidelines which can lead to quality variation among healthcare providers (22). Telehealth has been previously used in the fields of mental health, and chronic diseases such as: asthma, Chronic Obstructive Pulmonary Disease (COPD), diabetes, cardiovascular diseases, and hypertension (1, 3, 23). It also recently demonstrated success in the management of mild to severe COVID-19 cases in China (2).

Although it is novel and innovative, telehealth still needs to be deployed in a manner that assures the attainment of quality standards that were required when resorting to traditional face-to-face interactions. This is particularly relevant to the COVID-19 times, which required, at some point in time, exclusive reliance on telehealth. There are several studies that refer to the quality of telehealth (24). None of them, though, tackle it from the macro perspective that has been suggested by international organizations such as the IOM and WHO. Telehealth quality was first assessed in 1996 in a telepsychiatry setting by assessing its accessibility and cost effectiveness (25). Both of those attributes of the received quality of care are important but do not cover for all aspects of the experience (24, 26).

Several studies have assessed healthcare workers' perception of the application of telehealth for patient care (27, 28). However, very few studies capture the patients' perception about their experiences, and none of them refer to evaluation tools that are contextualized to an emergency in the Middle East and North Africa (MENA) region. Therefore, the aim of this research study is to develop and validate a tool, and to deploy it to explore the patients' perception of and satisfaction with the utilization of telehealth services in Dubai, UAE during the COVID-19 pandemic. Accordingly, in this study we strive to address the following research questions:

1. How do patients perceive the quality of the telehealth consultation experience?
2. How satisfied were the patients with the quality of their telehealth consultation, and what variables were associated with patients' satisfaction?
3. What meta-inferences can be derived upon merging the findings of the qualitative analysis (i.e., perception of experience) with that of the quantitative one (i.e., extent of satisfaction, and the interplay across variables)?

## Methods

### Context of the Study

The study was undertaken in Dubai, UAE. It is estimated that around 30% of the healthcare facilities in Dubai are public and the remaining 70% are private (29, 30). This research study was conducted in three multidisciplinary hospitals and nine community clinics of an international private hospital group: Mediclinic International. This hospital group has two operating platforms other than the one in the UAE: South Africa and Switzerland. It also has shareholding in Spire Healthcare- a United Kingdom-based healthcare group (31).

### Responding to COVID-19

The UAE took numerous steps to curtail the spread of COVID-19 including a lockdown of public places, such as shopping malls, with a set curfew. After the first case was detected in the UAE on 29th January 2020 and as the infection rate was rapidly escalating, Dubai Government instigated a sterilization campaign on 26th March 2020 as an effort to contain COVID-19. This involved mass sanitization of streets and public places. As part of the campaign, restrictions to movement have been implemented with a night curfew. As such, members of the public were prohibited from leaving their homes from 8:00 p.m. to 6:00 a.m. except for essential needs (32, 33). As the number of COVID-19 cases decreased, the restrictions lessened, and public places reopened slowly. However, new social distancing regulations were established in public spaces including restaurants and public transportation (34).

The “Dubai COVID-19 Command-and-Control-Center” (C^∧^3) was established to enhance collaboration across the healthcare sector and ensure alignment with the Dubai Government's efforts to tackle the COVID-19 outbreak (35, 36). Strict regulations for the healthcare workplace were put into effect. The directives generated by the C^∧^3 included obliging patients who need to undergo an aerosol generating procedure to have a negative COVID-19 Polymerase Chain Reaction (PCR) nasal swab. Healthcare workers were required to wear Personal Protective Equipment (PPE) to ensure the highest level of safety and protection to the patients and the healthcare workers. Also, telehealth was introduced to the healthcare system and heavily used and recommended for patients limiting person-to-person interaction and hence reducing possibility of virus transmission. The use of telehealth was most useful and needed during the lockdown especially for patients who need continuous follow-up consultations and medication refills. Telehealth also served as a mode of education providing virtual teaching for students in the healthcare sector, including medical and nursing students (37).

### Research Design

**Figure 1:**
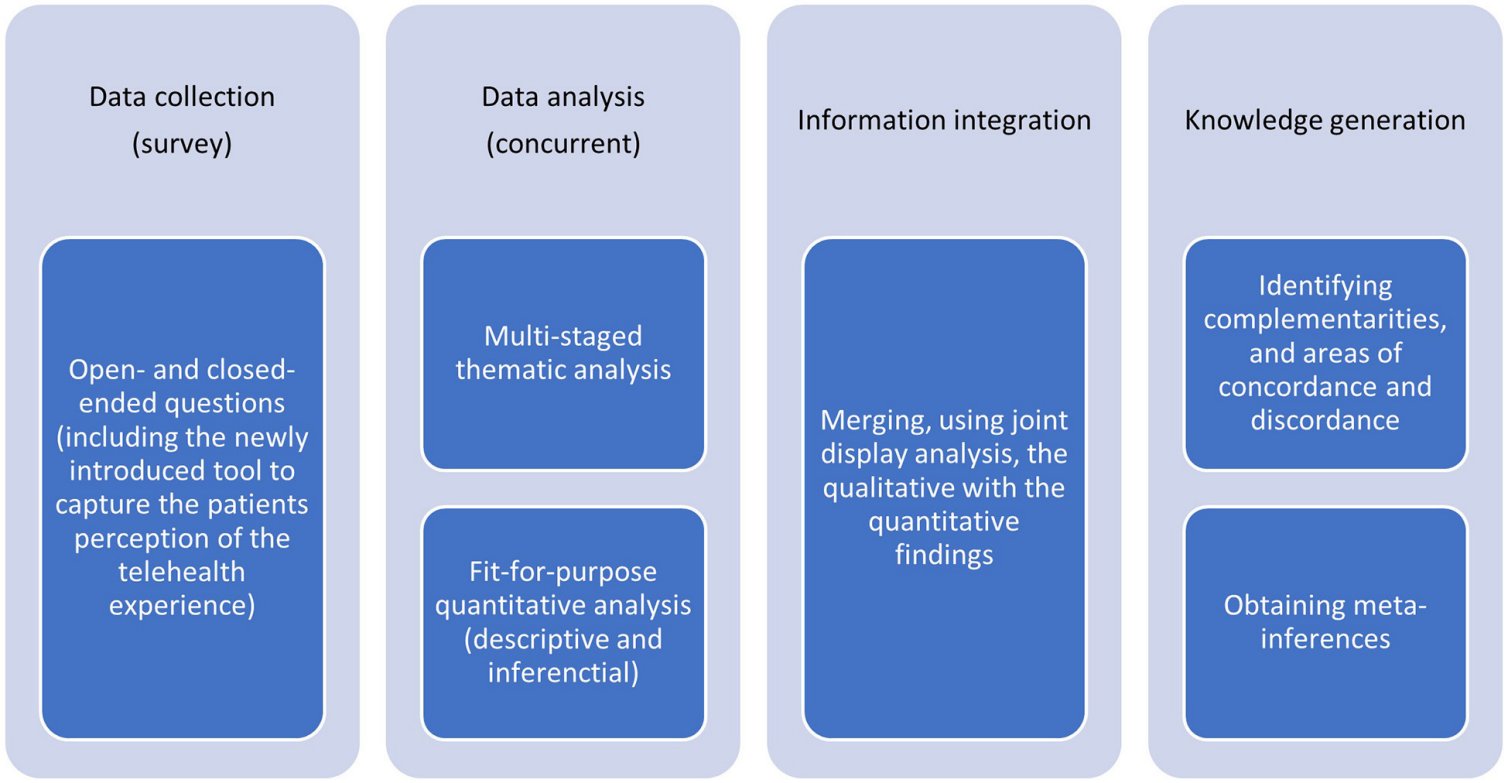
Four sequential stages of the convergent mixed methods research design adapted for this study.

The study's ethical approval was granted by the Mohammed Bin Rashid University of Medicine and Health Sciences (MBRU), Institutional Review Board (Reference # MBRU-IRB-2020-028). A multi-phased convergent mixed methods study design (38–41) was adopted, as demonstrated in Figure 1, to systematically, from a macro perspective, understand the patients' perceptions regarding the quality of their experience with telehealth medicine.

This study was characterized by four sequential phases: data collection, data analysis, information integration, and knowledge generation. In the first phase, the qualitative and quantitative data were concurrently collected. The second phase of this study involved analyzing the quantitative datasets independently from the qualitative datasets. Following that, we integrated the generated information, comparing and relating findings from the independent parallel analyses. The information integration, which is expected to raise the validity of the study's findings, relied on joint model analysis (38, 42). The interpretation of the integrated information led to the generation of knowledge, which constituted the last phase of the research design.

### Data collection

#### Tool Formation

The data was collected using a survey that was designed specifically for this study. This survey aimed at assessing the perception of patients regarding their telehealth experience during COVID-19 in Dubai, UAE. The researchers were also interested to investigate the patients' point-of-view regarding the usability and sustainability of telehealth.

The survey was developed through the joint efforts of six researchers (AAM, EAM, FO, GAA, MA, and RAQ). The construction of the survey took place over five stages: literature and desk reviews, selection of relevant questionnaires, validated data collection tools, modification and integration of elements of the selected tools, content and face validation of the tool, and questionnaire translation.

##### Literature and Desk Reviews

A thorough literature review was conducted to identify validated telehealth surveys that acquired information on patient satisfaction and usability of telehealth services.

##### Selection of Relevant Questionnaires

Two of the retrieved tools stood out: the MinuteClinic questionnaire (43) and the Telehealth Usability Questionnaire (TUQ) (44). Both tools were retrieved from recently published peer-reviewed articles.

##### Modification and Integration of Elements

Specific elements were identified from the selected tools, which were in turn contextualized to fit the intricacies of the situation under investigation. The researchers also complement the closed-ended questions with qualitative, open-ended ones to match the selected research design which was meant to have an exploratory component (along with the investigative one). This also contributed to setting the developed tool apart from existent ones since all those that were identified by the researchers solicited for quantitative data only. As such, the tool, generated as part of this research study, was composed of four segments: patient's sociodemographic information, respective consultation overview, perceived quality of experience, and perceived future usefulness and utilization of telehealth (Appendix 1 in Supplementary Material).

The first segment of this survey inquired for the patient's sociodemographic information which included gender, age, highest level of education completed, and nationality. The second segment of the survey was meant to shed light on the specificities of the telehealth consultation. It inquired whether, or not, the respective consultation was covered by the insurance. It also asked for the healthcare facility that the teleconsultation took place in conjunction with, the type of telehealth consultation (audio or video), reason for choosing telehealth consultation, and the purpose of the telehealth consultations. It also identified the medical specialty of the physician whom the patient tele-consulted with. The same segment also investigated any potential concern(s) that the patient had prior to the teleconsultation, and inquired about the level of satisfaction with the services delivered (relative to reaching out to the physician directly and to the traditional, face-to-face consultation), along with checking whether, or not, the patients perceived the experience as confusing and/or complicated. As for the final part of this segment, it asked for an approximation of the consultation duration.

The third segment composed the core of the survey measuring 14 components of the quality of the services, as per Table 1, against a Likert-type scale of five points (1: Very Dissatisfied, 2: Dissatisfied, 3: Neutral, 4: Satisfied, and 5: Very Satisfied).

**Table 1:** Outline of the third (main) segment of the survey.

**Components**
Access to telehealth consultation
Availability of preferred physician
Ease of booking an appointment prior to the consultation
The preparatory support that you got, from Mediclinic Middle East, prior to the consultation
Waiting time for the consultation
Ease of remotely seeing the physician during consultation
Ease of remotely hearing the physician during consultation
Ease of seeing any images on the monitor during the consultation
Ease of engaging with the physician during consultation
Communication with the physician during consultation
The extent to which the physician addressed your questions and concerns
The treatment plan and patient educational materials you received
The physician's performance/ability to identify and address your health problem
The overall quality of care received during the consultation

As for the last segment of the survey, which inquired about the sustainability and future use of telehealth. It measured several aspects (such as likelihood of the patient resorting to telehealth again and recommending it to others) against a Likert-type scale of five points. The qualitative section of this segment included three open-ended questions that explored strengths, weaknesses, and opportunities of improvement of the telehealth consultations. In contrary to the closed-ended questions (which were mandatory), the open-ended ones were optional.

##### Questionnaire Validation

The generated data collection tool underwent two validation phases. Firstly, experts in the subject matter were contacted for the content validity. These experts included a current practicing physician, a medical school faculty, and a health services researcher, all of whom were well-informed about the UAE healthcare sector, in general, and the utilization of telehealth in the UAE, in specific.

Secondly, the survey was disseminated to 10 randomly selected members of the community of a medical university in Dubai, UAE, who had consulted at least once with the facilities under investigation, to assess the readability and comprehensibility of the questions and the sequence by which they are presented (i.e., face validity).

##### Questionnaire Translation

The questionnaire was translated into Arabic which is a widely spoken language in Dubai, UAE.

#### Participants' Recruitment

A total of 100 randomly selected patients were invited to participate. Participation in this data collection initiative was completely voluntary. The privacy and the data confidentiality of the patients were protected, and no personal identifiers were recorded. The survey was open for participation from 16th September 2020 until 23rd December 2020.

#### Survey Administration

A third party at the administration office of the Dubai branch of the international chain of private hospitals and community clinics sent-out emails inviting 100 randomly selected patients, who tele-consulted at any of the multidisciplinary or community clinic units during the COVID-19 pandemic period (March through December 2020), to participate. The electronic survey (assembled via Google forms platform) was shared via a link embedded in the emails sent-out by the administrator.

### Data Analyses

#### Qualitative Analyses

The qualitative data analysis started after the conclusion of the data collection phase. All the qualitative data, collected from the respective survey (i.e., the three corresponding open-ended questions), was analyzed using thematic analysis by six researchers (AAM, EAM, FO, GAA, MA, and RAQ). One of those researchers is trained in qualitative socio-behavioral research, and handled the responsibility of controlling for the consistency of the analysis performance of the rest of the coders, who were divided into two groups. The subjectivity of the researchers was recognized, right from the start of the analysis, to avoid affecting the integrity of the qualitative analysis trajectory. Each of the two groups independently analyzed the qualitative data. Prominent patterns were identified after thorough examination of the datasets. The process was inductive, based on constructivist epistemology. The process of analysis followed the six-step framework initially introduced by Braun and Clarke (45–47). NVivo software version 12 plus (QSR International Pty Ltd, Vic, Australia) was used to code the data, and in turn expedite the categorization of the relevant text fragments.

The analysis process started with the researchers familiarizing themselves with the data. Following that, initial codes were generated. Then, the researchers searched for themes, and thoroughly reflected upon them, in their assigned groups. Both groups of researchers, then, convened to discuss their respective schemes and in turn reach a consensus on the best means of categorizing the data segments. They then progressed, as one entity, to defining and naming these themes. The output of this collective exercise constituted the basis of the study's conceptual framework which guided the last step of the adapted analysis technique, where the researchers reported upon the findings in accordance with preset recommended guidelines (39, 45).

#### Quantitative Analyses

The quantitative data was analyzed using SPSS for Windows version 27.

##### Descriptive Analyses

The frequencies of all the categorical variables were calculated. For the components of the tool in the third segment of the survey, an overall score of satisfaction was calculated. In addition, for each of the components, independently, and the score of satisfaction for each patient, the mean and standard deviation were calculated.

The tool used for capturing the perception of the patients was tailor-made for this study. The validity tests of Cronbach's Alpha, and the Principal Component Analysis (PCA) of the Kaiser-Meyer-Olkin (KMO) and Bartlett's test were performed to ensure internal consistency and check external variance, respectively.

For the inferential analyses, to select the appropriate tests, a test of normality was conducted for each of the variables under investigation, including but not limited to the components of the tool and the overall score of satisfaction. The data points of all the variables, with no exception, turned out to be not normally distributed.

##### Inferential Analyses

Since the variables data turned out to be not normally distributed, a matrix of bivariate correlations was developed using Spearman test to assess the extent to which the overall score of satisfaction can be explained by changes in the patients' perception of the components of the score. Mann-Whitney test was used to compare the overall score of satisfaction, and each component (across the patients) independently, between categories of dichotomous variables (e.g., Gender, and Whether, or not, the consultation was covered by the insurance). As for the variables that are characterized by more than two categories (e.g., Age, Education, and Nationality), the Kruskal- Wallis test was conducted to assess their association with the overall score of satisfaction and with each component of the tool in the third segment of the survey.

#### Mixed Methods Integration

The findings from both types of analyses (quantitative and qualitative) were merged through mapping them onto each other and carefully reflecting upon them. This mixed methods integration was done using an iterative joint display (i.e., meta matrix) analysis process to promote methodological rigor which ultimately led to meta inferences (48, 49). This analysis was guided by the conceptualization of quality of health care as key elements of the experience that come together to create a whole that is more than the sum of its parts. These elements according to several international entities, such as the World Health Organization (WHO) (50), include: safety, patient-centeredness, effectiveness and efficiency, timeliness, and access and equity (51).

## Results

Out of those 100 patients, 94 responded (i.e., response rate = 94%). Each of the 94 participants was given a unique identification number (i.e., 01 through 94). Out of the 94 participating patients, 53 responded to at least one of the three optional open-ended (qualitative) questions of the fourth segment of the survey.

### Qualitative Data

**Figure 2:**
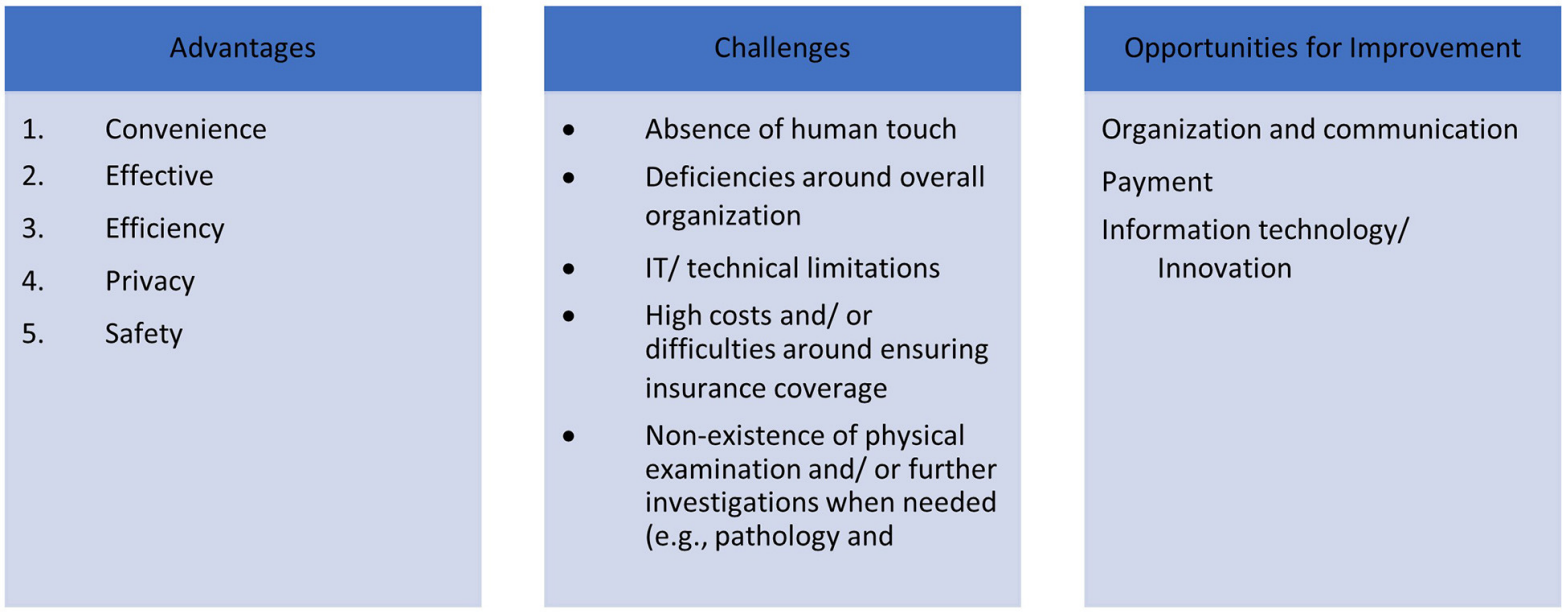
Study's conceptual framework (illustrating the categories and the themes that emerged from the qualitative analysis).

The thematic analysis resulted in three themes, revolving around the quality of the patient telehealth experiences: “Factors that worked to the benefit of the patients” (i.e., advantages), “Factors that the patients were not in favor of” (i.e., challenges), and “Opportunities for improvements as perceived by the patients” (Figure 2). Within the “Factors that worked to the benefit of the patients” theme, five categories surfaced: Convenience, Effectiveness, Efficiency, Privacy, and Safety. The “Factors that the patients were not in favor of” theme encapsulated five other categories labeled as: absence of human touch (i.e., physical contact and depth of the patient-physician connection/interpersonal rapport), deficiencies around overall organization, IT/technical limitations, high costs and/or difficulties around securing insurance coverage, and non-existence of physical examination, and (when needed) further investigations (pathology and laboratory medicine). As for the “Opportunities for improvements as perceived by the patients” theme, it included three interlinked categories: organization of the experience with emphasis on communication as a cornerstone, payment, and the information technology and innovation.

#### Theme 1: Factors That Worked to the Benefit of the Stakeholders (i.e., Advantages)

This theme refers to the strengths of the telehealth medicine, as perceived by the patients.

##### Convenience

As part of this category, the patients indicated that they appreciate the availability of this alternative. They emphasized the ease-of-access to healthcare as an added value to their experience:

02: “…I value that I can consult a physician without the need to visit the hospital…”
43: “…it was great, I was able to talk with the doctor without the need to travel to him…”

The versatility of the telehealth was also brought-up:

71: “…I consulted with my physician from the comfort of my home…”
78: “…you can consult your doctor from anywhere in the world…”

The patients also noticed that telehealth offered them some advantages over face-to-face consultations:

01: “…it was simpler for the physician to follow-up on and discuss my condition virtually…”
05: “…telehealth made consulting with the physician easier. Before COVID-19, physicians were only available in the outpatient clinics during their working hours which required that I take time off from work to come for my appointments. With telehealth, the physicians became more accommodating…”

##### Effectiveness

In this category, patients expressed how the telehealth medicine enabled meeting the consultation's preset targets. Some patients mentioned that they were satisfied with the performance of the physicians and their support staff online.

01: “…I am very impressed with my doctor and his team at the hospital…”

A lot of the patients perceived their experience as successful, and some viewed this set-up to be even better, in meeting the consultation's preset targets, relative to the traditional options (i.e., face-to-face consultation or that over a regular phone call).

18: “…I found it to be richer, where the physician was more informative than over a regular phone call…”

This particularly applied to follow-up consultations and those aimed at medication refill, where patients recommended to sustain telehealth for follow-up appointments.

24: “…I recommend teleconsultation especially for follow-up appointments…”

They also expressed appreciation of this alternative for medication prescription and other minor consultations:

33: “…most of my consultations are just to refill my medicines, so there is no need for face-to-face visits…”
37: “…if you do not require a physical examination, this is a very good means to get the job done…”

Some patients also felt that their physicians were more engaged with them virtually relative to face-to-face consultations.

59: “…my physician is more engaged online…”

##### Efficiency

Patients, as part of this category, described ways in which this alternative approach to healthcare provision saved resources. The patients noted that the preset targets were attained with less resources. Some patients highlighted how a lot of traveling and waiting time, and costs are saved, and traffic is avoided.

03: “…it is fast and simple. It saves commuting and waiting time, and costs. It is much more efficient…”
29: “…there is no waiting time in reception, and no driving to a clinic. It is so quick…”
54: “…this alternative is fantastic for those of us with limited time who do not need to physically attend…”

##### Privacy

Some patients expressed appreciation that this method reassured them that their confidentiality and privacy were maintained.

75: “vprivacy for patients like me who do not want others to know about their medical journeys…”

##### Safety

It was clear to the patients that this alternative to face-to-face consultation is much safer, and reduces the chances of exposure and in turn the risk of infection. This seemed particularly important to the patients given the individual and collective fear associated with the pandemic, and the directives around social distancing.

15: “…social distancing was maintained…”
44: “…it enabled consultation without physical contact which prevented further spread of disease…”
69: “…it is useful for critical situations like COVID-19…”

#### Theme 2: Factors That the Stakeholders Were Not in Favor of (i.e., Challenges)

This theme refers to the weaknesses and difficulties of telehealth medicine, as perceived by the patients, and the struggles that they faced during their virtual consultations.

##### Absence of Human Touch

The patients noticed the absence of physical contact, and how it affected the depth of the patient-physician connection keeping the relationship transactional in nature.

15: “…physicians seem less interested and concerned in patients' problems when they are offering their consultation online…”
58: “…for follow-up appointments, it is fine. For a first-time visit, I prefer in-person consultation…”
68: “…I miss interacting face-to-face with my physician…”
72: “…I prefer to see a physician in person…”
77: “…there is no physical contact- the physician is unable to physically check the patient…”

The patients highlighted that this communication medium, relative to face-to-face interactions, limited the capacity to develop a human connection which appears to be key, in their opinion, for building interpersonal rapport.

05: “…I would not like to have a teleconsultation for a new complaint, especially if I have not had face-to-face interactions with the respective physician. Teleconsultation makes health care business-like; the focus seems to be on swiftness. The conversation becomes limited to the problem-at-hand. There is more to health and wellbeing than the symptom…”
17: “…nothing beats face-to-face interactions, please do not try to convince me otherwise…face-to-face is always preferable, irrespective of the reason for consultation…”
19: “…after all, face-to-face interactions are way more humane, and enable exercising empathy and contributes to building rapport…”
81: “…any new physical development may go unnoticed; physicians may miss signs. Physical examinations are a must sometimes…”

##### Organizational Deficiencies

Some patients complained about how the adoption of telehealth suffered from some organizational deficiencies. Some of the concerns were around challenges that the patients received prior to the actual consultation:

39: “…booking an appointment is quite difficult…”

Other concerns were related to the processes that needed to happen after the consultation. The patients raised a lot of concerns related to the billing and collection process.

07: “…the payment logistics were problematic. They stressed me out; I kept on receiving payment requests, although I had settled the payment soon after my consultation was over…”

Suggestions to simplify the process around settling payment was frequently alluded to:

38: “…the payment process is unnecessarily complicated. For example, the email is not arriving at my Yahoo inbox even though other emails are. It would be better to receive the message via WhatsApp…”
59: “…the billing process can be improved; it can be simplified. There is an unnecessary delay between finishing the consultation and receiving the invoice…”
78: “…the process of settling the payment after the consultation needs to be simplified. It takes quite some time to receive the payment link. My physician goes out of his way to help me out, but the assigned administrative staff is not doing a good job…”

##### Information Technology/Technical Limitations

A few of the patients referred to technical glitches that they experienced during their consultations.

07: “…technical issues during the remote session affected the quality of my experience; also, we needed to make-up for those glitches which increased the duration of the consultation…”
18: “…I faced technical problems. I am not sure if it was from my side or that of the physician. The video was not clear…”

##### High Costs and/or Difficulties Around Ensuring Insurance Coverage

Some patients seemed to believe that this alternative should not be charged equally to the face-to-face consultation.

26: “…disproportionate costs: what we receive online should not be charged like traditional, full-fledged visits…”

Also, some insurance companies did not approve to cover the expenses of telehealth consultation.

07: “…not covered by my insurance. I do not know why: so many things are not making sense nowadays. Afterall, it is the same service with the same physicians in the same facility. The only difference is that I cannot come to the clinic and that is why I am needed to consult virtually…”

##### Non-existence of Physical Examination and/or Further Investigations (e.g., Pathology and Laboratory Medicine)

The patients, especially those requiring physical examination, believe that telehealth consultation lags in terms of effectiveness.

32: “…what would I do if I have a physical issue that my physician needs to look at? Phone or video calls would not do…”
37: “…if the physical symptoms need to be examined this could be a challenge…”
55: “…your physician cannot examine you physically, as simple as that…the consultation remains suboptimal…”

The sufficiency of this medium of communication seemed questionable to some patients:

03: “…telehealth works only if the patient is not feeling any physical pain or symptom that requires a medical physical examination/diagnosis…”
54: “…specific symptoms cannot be detected virtually; you need physical examination. Otherwise, these symptoms will go unnoticed…”
56: “…if you have critical health problems, then you should go to hospital and see a doctor…”
70: “…In my opinion, there are many illnesses where physical examination of the patient gives valuable information to the physician about the symptoms/actual illness. In such cases telehealth consultation will not be sufficient…”
71: “…I see how for a physical examination, telehealth may not be a good enough of an idea…”

Some patients also referred to the more proactive role that they had to play to make-up for the gap, and the entailed challenges, due to the transition to the online environment.

60: “…the effectiveness of the consultation relies heavily on the patients. Patients have a bigger responsibility relative to face-to-face consultations, since they have to be able to accurately describe their conditions, concerns, and symptoms…it is not easy…”

Besides that, the patients also emphasized how the crucial process of diagnosis is absent virtually.

29: “…what if one got appendicitis, just like what I had to go through in 2019, or if blood tests and x-rays are required. The results of those tests aid in getting an accurate diagnosis, such as taking one's temperature, measuring one's blood pressure… telehealth has been useful but is very limited…”
82: “…if the medical team requires additional information such as pulse, blood pressure, and so on. Most people do not have the equipment at home to provide this information…”

Some patients viewed telehealth consultation to impede continuity of care when further investigations are needed.

21: “…had I been in the hospital, I would have easily gone for the x-ray that was requested by the physician. I do not know what to do now; I am waiting to hear back from the assigned administrative staff. I wonder if I will be allowed to go to the hospital…”
25: “…if further diagnosis (laboratory test or so) is required, one needs to go to the clinic or hospital; it is a hassle. It would have been smoother to just be there and get through all that needs to be done in one chunk…”
40: “…some things cannot be done over the phone. The physician asked me to come in. Who knows when I will be able to come in? What if something happens to me prior to then? I wish I was at the hospital right now…”

#### Theme 3: Opportunities for Improving Patient Experience

This theme refers to the opportunities that the patients perceived to be worth acting upon to improve the quality of the telehealth consultation experience.

##### Organization and Communication

This category encapsulated the points that the patients made in relation to optimizing processes around the experience, including scheduling for appointments, and means of effectively following up.

01: “… it is difficult to reschedule an appointment…”
61: “… to ease the process from appointment until getting the payment link, my doctor helped out, but the staff responsible for following up did not do a good job…”

The most prominent opportunities for improvement, identified by the patients, were around communication.

43: “…communication and promptness- if an emergency arises in which the doctor cannot make the appointment, the nurse needs to let the patient know rather than leaving them wondering what is happening…”

Most of the lags in communication, according to the patients, were prior to the consultation.

05: “…the call center operator informed me that someone will email me back, but I did not receive anything afterwards…”
07: “…the date and time should have been better communicated to me…”
71: “…better prepare the patient prior to the consultation as a means of managing expectations…”

##### Payment

The patients' suggested reducing the entailed costs. This idea was frequently alluded to by patients with no medical insurance:

02 “…the cost of telehealth consultations should be reduced…”

The patients also highlighted the importance of enhancing the processes around settlement of payments (i.e., billing and collection).

03: “…the payment process should be more practical…”
33: “…the payment process could be easier…”

##### Information Technology/Innovation

Several patients referred to the potentiality of leveraging IT and innovation for the betterment of the telehealth consultation experience. For example, the patients were concerned about the lack of recording vital signs. Hence, they recommended developing a solution that would enable them to record their vitals from the comfort of their homes.

29: “…possibly link the teleconsultation to an application that does the needed measurements…”
48: “…advanced technology may be utilized to allow for distance assessments that the patient can perform and share with the medical team just before or during teleconsultation…”
82: “…the teleconsultation may be linked to certain new medical technologies to provide the medical team with needed information, such as real-time vital signs…”

Another opportunity for improvement was around the booking system. Some patients reflected upon solutions in that realm, as well.

90: “…creating a hospital application that shows you the availability of the respective physicians when booking appointments; you get several options to choose from…and once your appointment is booked, you get a detailed message via this application with all the details of your appointment…”

The patients also mentioned how technological devices can be adapted to improve the teleconsultation video quality.

18: “…advanced technological devices may be adopted to improve the video and quality of images throughout the teleconsultation…”

An online platform or a webpage linked to the hospital's website where patients are enabled to book their own appointments.

32: “…patients should be able to book appointments through an online platform that is linked to the hospital's system…”

The patients also identified an opportunity for improvement around the communication with the hospital, all of which is done via Short Message Service. Apparently, this service does not work effectively on all telephone operating systems, which is why a more reliable alternative needs to be put in place.

54: “…the text messages for appointments confirmations do not work on specific mobile phones operating systems, which becomes particularly problematic when the patient is requested to send back appointment confirmation texts…”

### Quantitative Analyses

#### Descriptive

**Figure 3:**
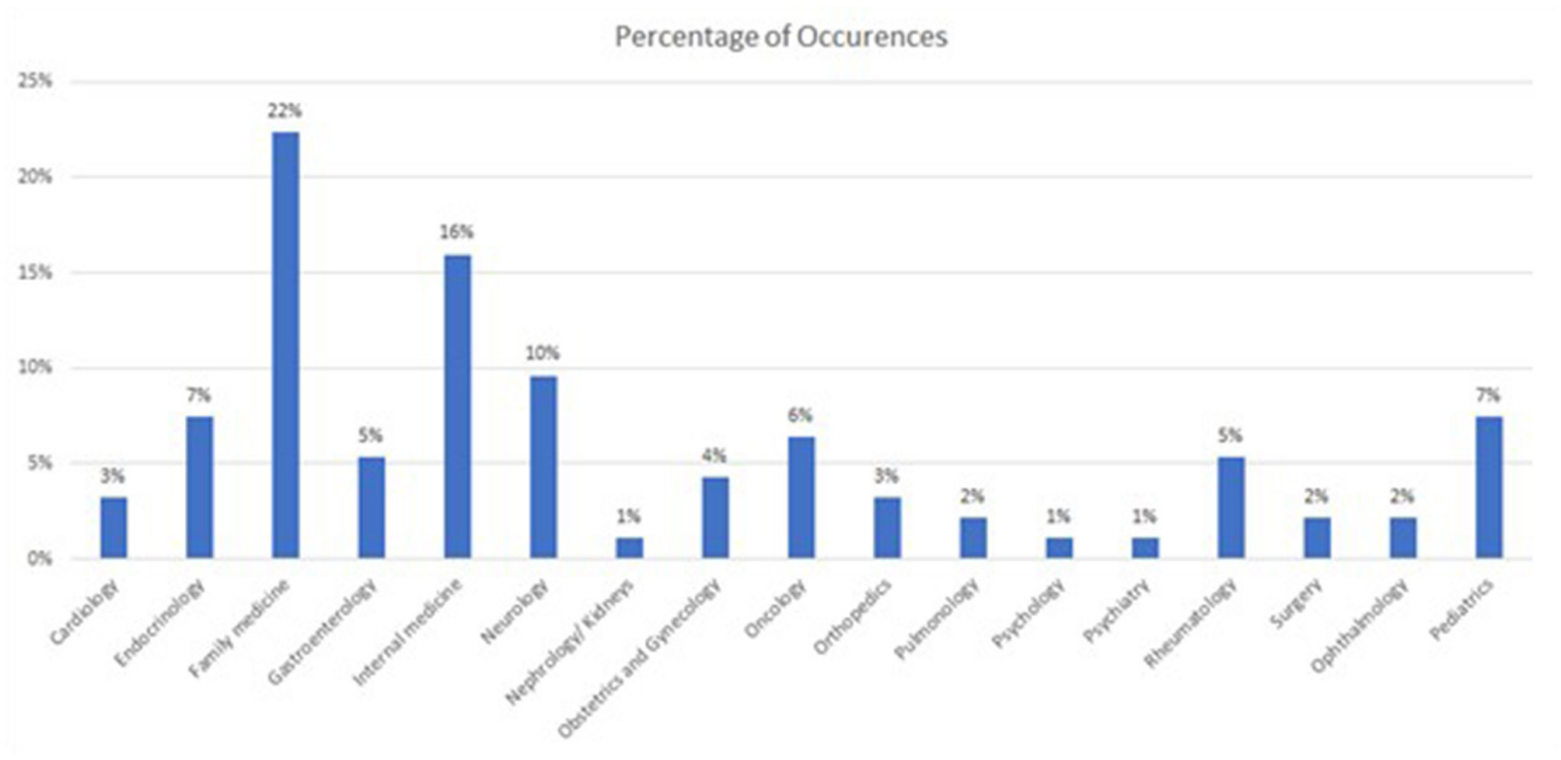
Distribution of patients, based on specialty sought.

The participants' sociodemographic information showed that the majority were female (58.5%). In terms of age range, 53.2% were between 36 and 55 years old, and 27.7% above 55 years old, 11.7% between 18 and 35 years old, and the rest were <18 years old. Moreover, most of the participants hold a university degree, with 42.6% with an undergraduate degree and 51.1% a graduate degree (e.g., Masters, Medical Doctor, or Doctor of Philosophy). Only 1 participant indicated elementary studies five participants selected high school as the highest level of education completed. In terms of nationalities, most of the participants were from India (20.2%), followed by the United Kingdom (18.1%) the United Arab Emirates (8.5%), and Egypt (7.4%). There were 5 participants from each of Pakistan, Philippines, Belgium, and the United States of America, 3 from South Africa, and 2 from each of Canada, Jordan, Netherlands, and Portugal. There was also 1 participant from each of the following countries: Australia, Bulgaria, Colombia, France, Germany, Ghana, Ireland, Italy, Lebanon, New Zealand, Sudan, Switzerland, Syrian Arab Republic, and Yugoslavia. The majority were covered by insurance (91.5%). The majority of the patients sought Family Medicine (22%) followed by Internal Medicine (16%), and Neurology (10%), as per the Figure 3.

**Figure 4:**
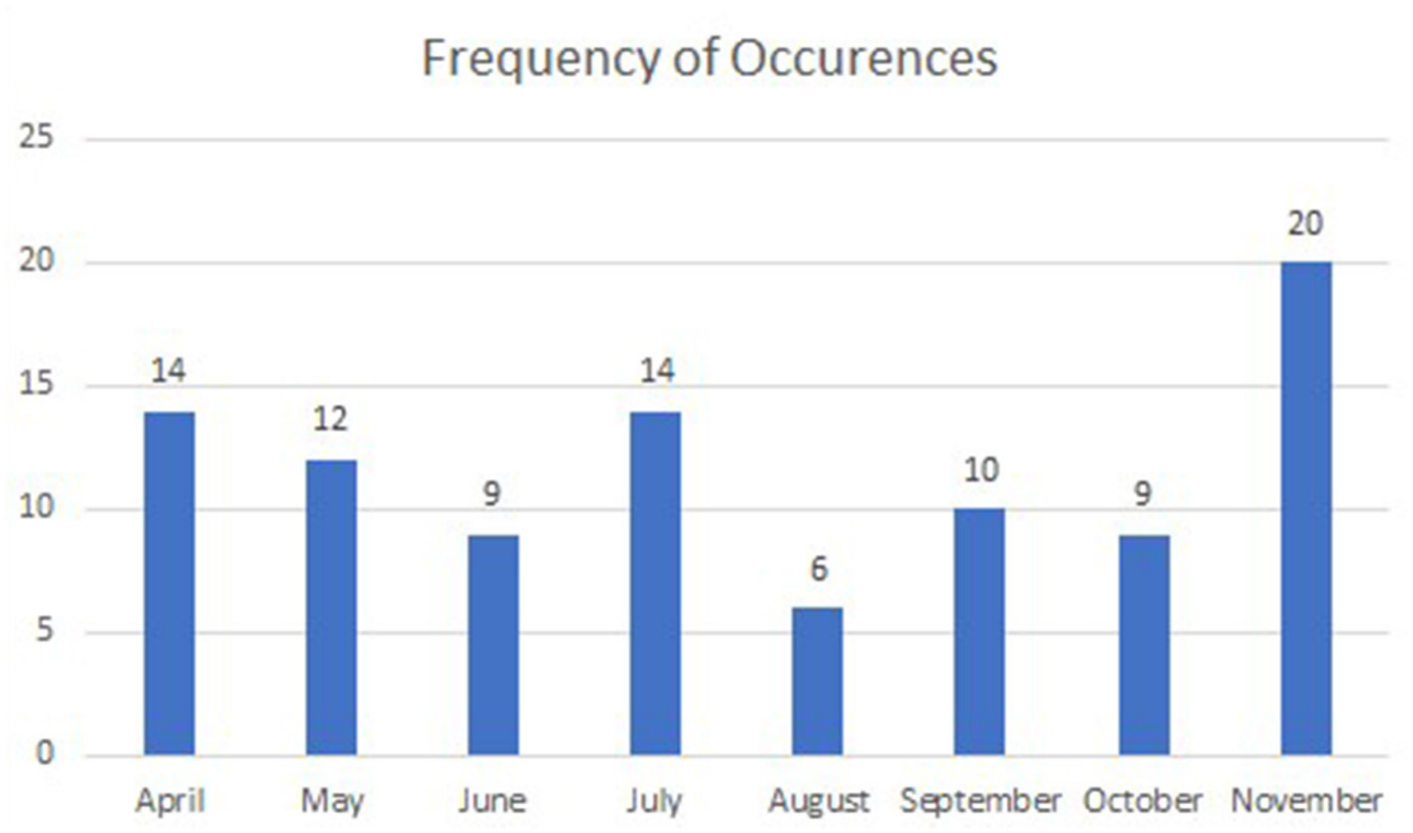
Distribution of patients, based on month of consultation.

In terms of the overview of consultations of the participating patients, the majority were covered by insurance (91.5%). Moreover, 59.6% of the patients received consultation from a physician who works in the out-patient clinics of one of the hospitals under investigation. As for the rest of the patients, they received consultation from physicians who work in polyclinics. All the 94 participants had received their consultation between April and November 2020, as illustrated in Figure 4.

**Figure 5:**
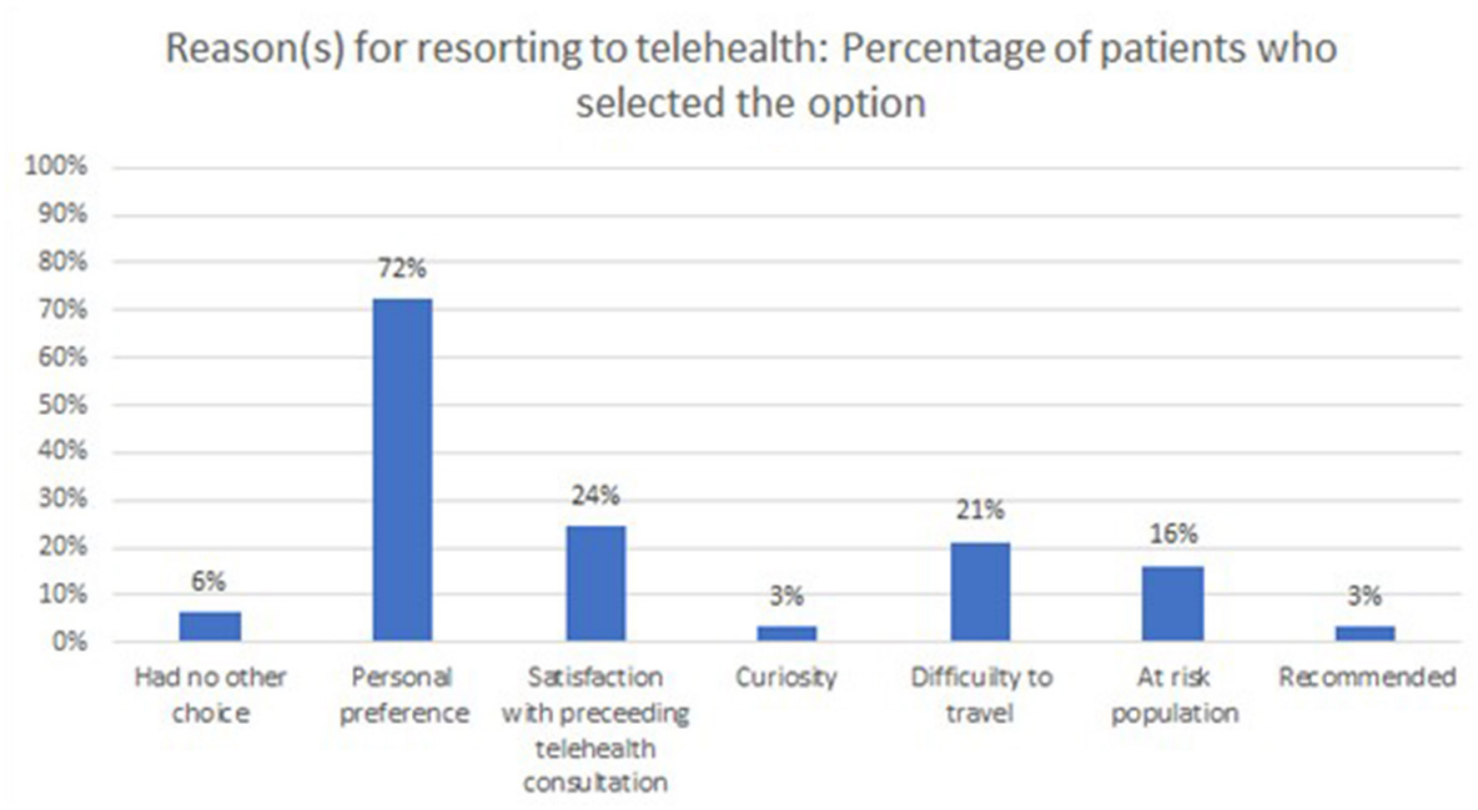
Distribution of patients, based on reason(s) for resorting to telehealth.

Most of the consultations were conducted over video-calls (77.7%) and the rest were audio-calls. The majority of the consultations took between 5 and 10 min (54.3%), followed by 15–20 min (33.0%), <5 min (10.6%), and finally more than 30 min (2.1%). The vast majority of the patients did not find anything about the telehealth consultation to be confusing and/or complicated (92.6%). Also, when the patients were asked to select all that applies in terms of reasons for resorting to telehealth, “personal preference” seemed to stand-out with 72% of the participants choosing it as one reason (on its own or among others), as illustrated in Figure 5.

**Figure 6:**
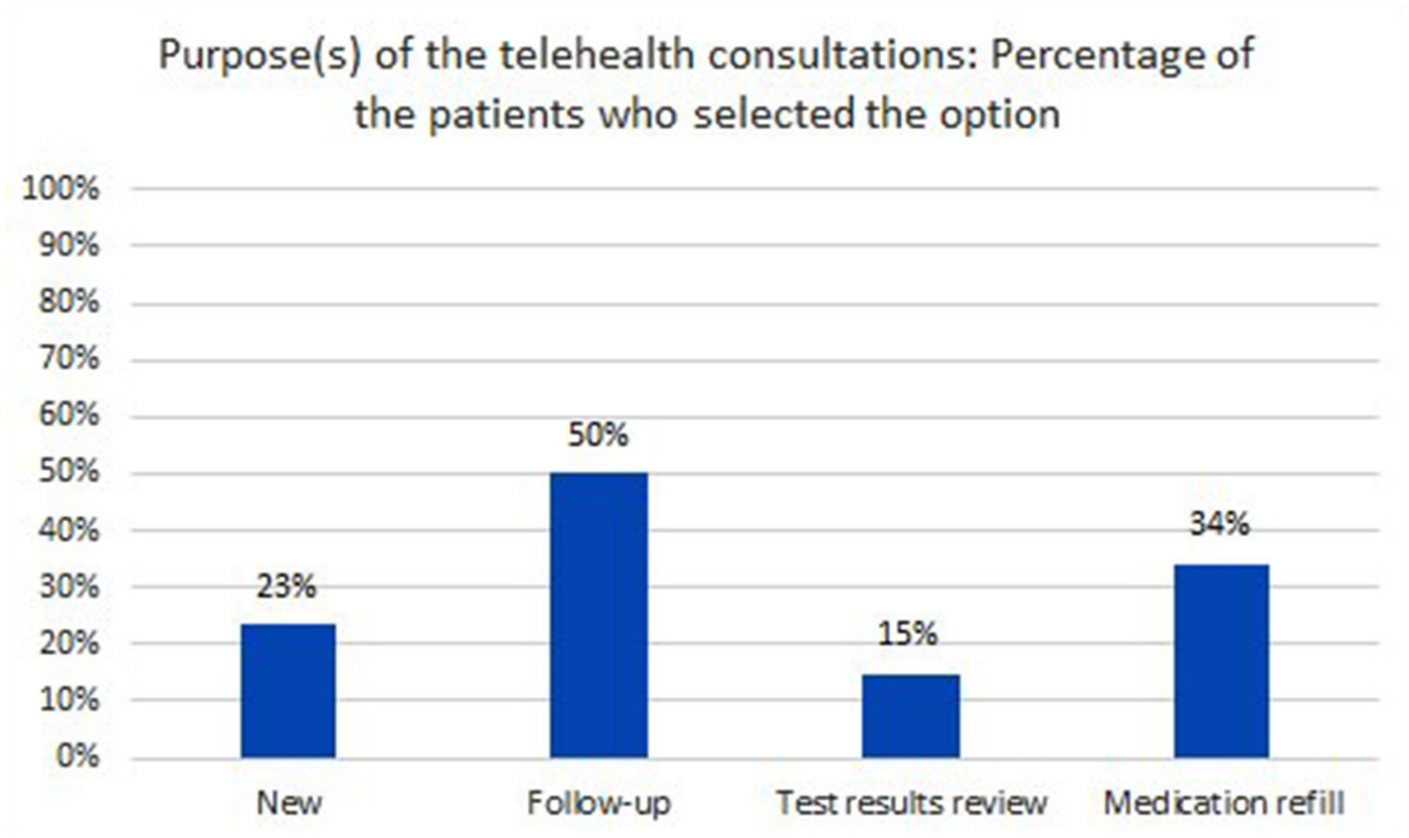
Distribution of patients, based on purpose(s) for consultation.

Moreover, when they were asked to select all that applies in terms of the purpose of the telehealth consultations, “follow-up appointment” seemed to stand-out with 50% of the participants choosing it as one purpose (on its own or with other), as illustrated in Figure 6.

**Figure 7:**
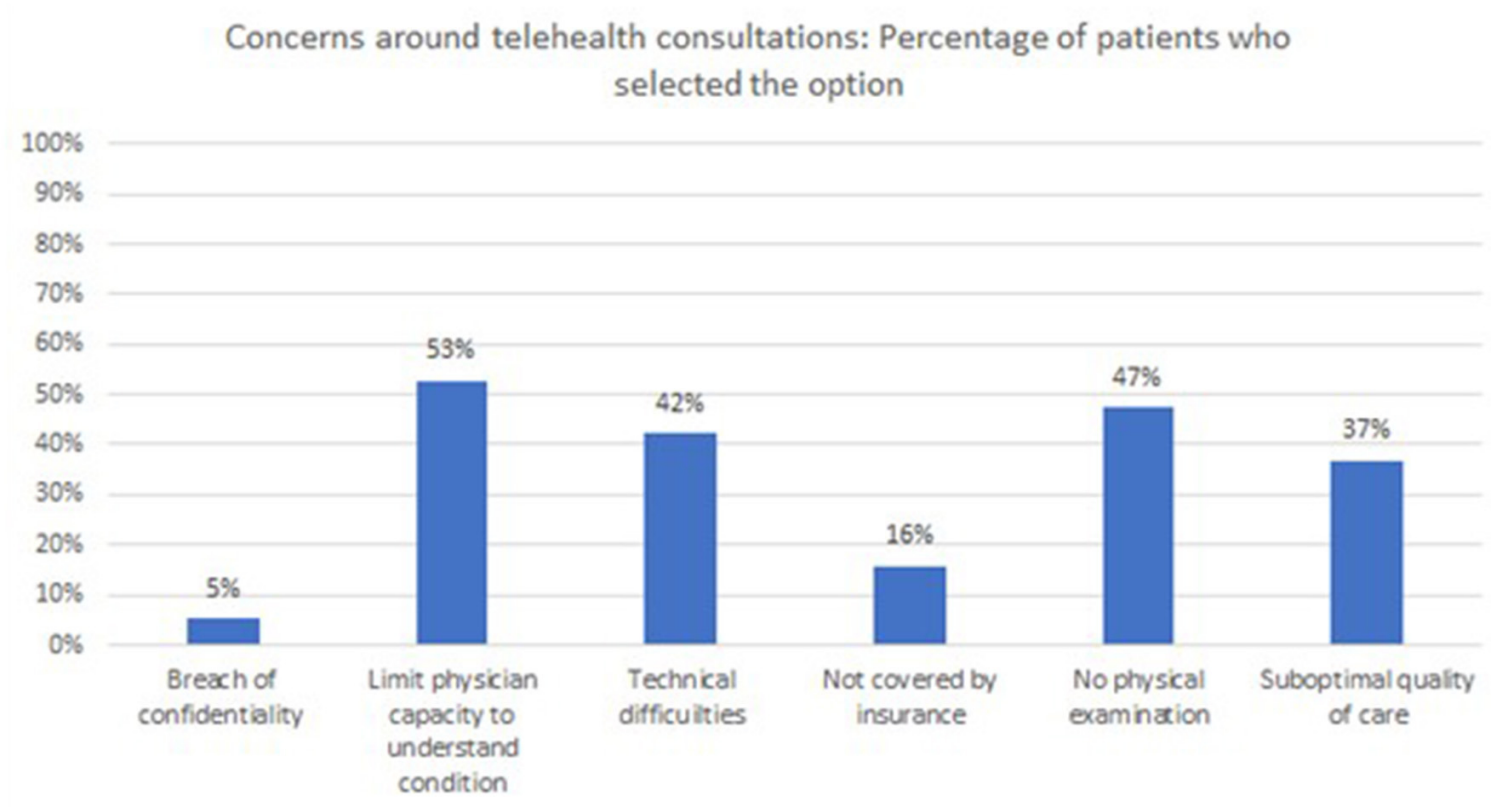
Distribution of patients (who had concerns prior to consultation), based on the selected concern(s).

Around 80% of the patients reported not having concerns or reservations regarding telehealth prior to the consultation. The concerns or reservations among the remaining 19 patients were distributed as per Figure 7.

Relative to directly reaching-out to their physicians (on their mobile devices), the participating patients' mean satisfaction turned-out to be 4.53(±0.83) with their most recent telehealth consultation. As for the mean of their satisfaction when compared to regular face-to-face consultation (i.e., in-person hospital visit), it turned-out to be 4.32(±0.93).

The reliability score of Cronbach's Alpha for the evaluation instrument that captured the perception of the patients was 98.9%. The percentage of the total average of satisfaction was 80.67%, as per Table 2.

**Table 2:** Output of descriptive quantitative analysis.

**Component**	**Mean ± SD**	**Percentage of the mean**	**Category**
• Access to telehealth consultation	4.37 ± 1.09	87.4	S-VS
• Availability of preferred physician	4.47 ± 1.04	89.4	S-VS
• Ease of booking an appointment prior to the consultation	4.28 ± 1.10	85.6	S-VS
• The preparatory support that you got, from the hospital group, prior to the consultation	4.16 ± 1.18	83.2	S-VS
• Waiting time for the consultation	4.29 ± 1.14	85.8	S-VS
• Ease of remotely seeing the physician during consultation	4.43 ± 1.07	88.6	S-VS
• Ease of remotely hearing the physician during consultation	4.53 ± 1.01	90.6	S-VS
• Ease of seeing any images on the monitor during the consultation	4.23 ± 1.17	84.6	S-VS
• Ease of engaging with the physician during consultation	4.35 ± 1.06	87	S-VS
• Communication with the physician during consultation	4.37 ± 1.06	87.4	S-VS
• The extent to which the physician addressed your questions and concerns	4.31 ± 1.17	86.2	S-VS
• The treatment plan and patient educational materials you received	4.35 ± 1.17	87	S-VS
• The physician's performance/ability to identify and address your health problem	4.32 ± 1.19	86.4	S-VS
• The overall quality of care received during the consultation	4.36 ± 1.20	87.2	S-VS
Score of satisfaction	56.47 ± 15.50	80.67	S

*S, Satisfied; VS, Very Satisfied*.

With a KMO close to 1, the sampling was determined as adequate. Also, according to the Bartlett's Test of sphericity, the null hypothesis got rejected with an identity matrix in which all the diagonal elements were 1 and all off-diagonal elements are 0. As such, the PCA (along with corresponding Eigenvalues) showed that 88.1% of the variance can be explained by the instrument, as a whole (*p* < 0.001). This means that the instrument is not only reliable (as per the abovementioned reliability score of Cronbach's Alpha) but also valid to measure what it is intended to measure.

The rest of the scale-type variables were all within the fourth segment of the survey, namely: perceived future usefulness and utilization of telehealth. As illustrated in Table 3, the mean of the likelihood of having patients resort to telehealth consultation at the same hospital group and recommend the experience was between Likely and Very Likely. Moreover, the percentage of the mean of the patients' perception of the likelihood of telehealth to become the primary means of consultation was 84.6%, while the percentage of the mean satisfaction if telehealth actually becomes the primary means of consultation was 77%.

**Table 3:** Output of descriptive quantitative analysis.

**Variable**	**Mean ± SD**	**Percentage of the mean**	**Category**
How likely are you to resort to telehealth Consultation at the hospital group again?	4.43 ± 0.85	88.6	L-VL
How likely are you to recommend telehealth at any of the units of the hospital group in Dubai to a friend or a family member?	4.53 ± 0.67	90.6	L-VL
How likely do you think telehealth will be used in the future as a primary means of consultation?	4.23 ± 1.07	84.6	L-VL
How satisfied would you be if telehealth became the primary means of consultation in the near future?	3.85 ± 1.22	77	S

*L, Likely; VL, Very Likely; S, Satisfied*.

#### Inferential

The score of satisfaction with the quality of the experience was not significantly different between male (59.00 ± 12.08) and female (54.67 ± 17.41) patients (*p* = 0.506). There appeared to be no statistically significant difference in the score satisfaction with the quality of the experience between those that got covered by the insurance and those that did not get covered. Also, the patients' extent of satisfaction with the quality of the experience appeared to not be associated with the reason why the patients resorted to telehealth consultation.

Nonetheless, the patients of the consultations that were follow-up were significantly more satisfied with the quality of the experience relative to those that were not (*p* = 0.003). Also, consultations that had medication refills as a purpose (on its own or with others) appeared to be significantly less satisfactory than the rest of the consultations (*p* = 0.039). As for the patients who were concerned about the quality of care of telehealth prior to the consultation appeared to be significantly less satisfied than the patients who did not have any related worries and also those that were worried but did not select this option as one of their concerns (p=0.010). The participants who consulted through a video-call, with a mean satisfaction of 58.01 (±15.12), rated the experience higher than those whose consultation was conducted via audio-call, with a mean satisfaction of 51.10 (±15.97) (*P* = 0.014).

There was a significant difference in the score of satisfaction with the quality of the service across the patients depending on their levels of education (*p* = 0.001). Where those who hold a postgraduate degree appeared to be more satisfied than those with only an undergraduate degree followed by those who had reached high-school and finally those that stopped at elementary or junior levels. In relation to age, the overall score of satisfaction showed borderline significance in association (*p* = 0.055), among the components of the tool, the only exception was: “The extent to which the physician addressed your questions and concerns” with a *p*-value of 0.021. The component: “Communication with the clinician during consultation” showed borderline significance with a *p*-value of 0.051. In all three cases, be it the overall score of satisfaction, or any of the identified components, the satisfaction level appeared to increase as the age increases. In terms of duration, patients whose consultation took 15–20 min appeared to be significantly more satisfied than the rest of the patients (p=0.001), followed by those whose consultation took 5–10 min, then those whose consultation took more than 30 min, and finally those whose consultation took <5 min. There was no statistical difference of scores of satisfaction across patients depending on their nationalities or the specialties that they sought.

#### Mixed Methods Integration

Mapping the findings of the thematic analysis onto that of the quantitative analysis uncovered a holistic perspective of the situation, illustrated in the study's side-by-side joint display (Table 4). The convergence of findings enabled the development of a thorough understanding of the patients' perception of the key aspects of the quality of the healthcare services that they received through telehealth during COVID-19. These aspects include: Safety, Patient-centeredness, Effectiveness and Efficiency, Timeliness, and Access and Equity.

**Table 4:** Output of the joint display analysis.

**Qualitative→**	**Meta-inferences**	**←Quantitative**
- Safety as an advantage	Safety	- Most patients did not have concerns regarding telehealth prior to the consultation. - The patients who were concerned about the quality of telehealth prior to the consultation appeared to be significantly less satisfied.
- Convenience and privacy as advantages - Absence of human touch as a challenge	Patient-centeredness	- Most patients selected “personal preference” as a reason for resorting to telehealth - Patients were satisfied with patient-centeredness (as defined by the components of the tool) before, during, and after consultation
- Effectiveness and efficiency as advantages - IT limitations and non-existence of physical examination and/or further investigations (when needed) as a challenges	Effectiveness and efficiency	- Most patients did not find anything about the telehealth consultation to be confusing and/or complicated - Relative to directly reaching-out to their physicians (on their mobile devices) and to regular face-to-face consultation (i.e., in-person hospital visit), participants appeared to be quite satisfied - “Follow-up appointment” was the most selected purpose for resorting to telehealth consultation - Follow-up patients were significantly more satisfied relative to those that were not - Medication refills patients were significantly less satisfied relative to those that were not - Patients who consulted through a video-call rated the experience higher relative to those whose consultation was conducted via audio-call - The more educated the patients are, the more satisfied they appeared to be (satisfaction: postgraduate degree > undergraduate degree > high school > elementary or junior school)
- Organization and communication, payment, and information technology as opportunities for improvement	Timeliness	- Most consultations took between 5 and 10 min (54.3%), followed by 15–20 min (33.0%), <5 min (10.6%), and finally more than 30 min (2.1%). - Patients whose consultation took 15–20 min appeared to be significantly more satisfied than the rest of the patients, followed by those whose consultation took 5–10 min, then those whose consultation took more than 30 min, and finally those whose consultation took <5 min. - Patients were satisfied with timeliness (as defined by the following component of the tool: waiting time for the consultation)
- High cost or difficulties around ensuring insurance coverage as a challenge	Access and equity	- Most patients were covered by insurance - Scores of satisfaction between those that got covered by the insurance and those that did not get covered were similar - Scores of satisfaction across male and female patients were similar - Scores of satisfaction across patients resorting to telehealth consultation for differing reasons were similar - Scores of satisfaction across patients of differing nationalities were similar - Scores of satisfaction across patients seeking differing specialties were similar - Patients were satisfied with access and equity (as defined by the following component of the tool: access to telehealth consultation waiting time for the consultation)

In terms of “safety,” the output of the qualitative analysis highlights it as a major advantage of telehealth medicine, especially given the persistently high risk of getting infected with COVID-19. Along these lines, the quantitative analysis revealed that most patients did not have concerns regarding telehealth prior to consultation, which is important because this variable appeared to significantly affect the patients' satisfaction with the experience. As for the “patient-centeredness,” convenience and privacy were identified as two major advantages in the output of the qualitative analysis. Moreover, the quantitative findings showed that patients were satisfied with the telehealth experience, which they favored over other alternatives. Yet, the absence of human touch was still highlighted by the patients as a challenge.

The codes: “Effectiveness” and “Efficiency,” surfaced (as is) in the qualitative thematic analysis, where IT limitations, and non-existence of physical examination and/or further investigations were brought-up as challenges. Similarly, in the quantitative analysis, patients were quite satisfied with the entailed processes, especially for follow-up appointments. However, the patients were not that satisfied with the experience when the purpose of their consultation was refilling medications. Their level of satisfaction was also significantly influenced by the patients' educational status. In terms of “timeliness,” the patients appeared to be satisfied with the waiting time. Yet, they identified opportunities for improvement around organization and communication, payment, and extent of leveraging existent IT. The ideal consultation duration, from the point-of-view of the patients, appeared to be 15–20 min. In relation to “access and equity,” although most of the consultations under investigation were covered by insurance, the patients' level of satisfaction was not affected by whether, or not, the consultations were covered. Their level of satisfaction was also independent from gender or nationality of patients, reason for consultation, medical specialty sought, which is also confirmed in their positive rating of the corresponding component of the tool. Yet, relevantly, high costs or difficulties around ensuring insurance coverage still surfaced among the challenges identified in the qualitative analysis.

## Discussion

The findings of the current study showed that the patients were quite satisfied with the quality of the telehealth consultation received, where the overall satisfaction rate was 81%. This finding is like that of a research study conducted by Dobrussin et al. during the COVID-19 era in a gastroenterology clinic. The respective study showed that the overall patient satisfaction rate with telehealth visits was >80% (52). Relevantly, another research work, which was carried out back in 2016, also found that the overall satisfaction rate, with all telehealth attributes, was substantially high (more than 94%) (52).

The current research study showed no statistically significant difference between the satisfaction amongst males and females. In addition, there was no association between satisfaction and whether, or not, the patients had insurance coverage. A few patients mentioned there were technical difficulties with billing and some patients believed the teleconsultation should not be as expensive as face-to face consultation especially that some insurance companies did not cover for the Telehealth consultation. Moreover, there was no significant difference in satisfaction across different specialties and races.

Interestingly, the current study showed a statistical difference in satisfaction among patients of different educational levels. Patients with postgraduate degrees were found to be more satisfied with the quality of teleconsultation than patients who only carry a high school diploma or elementary certificates. This might be because the more educated the patients are, the more acquainted they are with maneuvering through the online set-up. This comes in conjunction with the finding that as the age of the participants increases, their level of satisfaction appears to increase, especially when it comes to the communication skills of the physicians and the extent to which the patients' inquiries were effectively attended to. As such, age can be assumed to play a confounding role in the relationship between the patients' level of education and satisfaction. Similarly, another study stated that patients who completed a higher education degree were significantly more satisfied than those who do not pursue higher education (53).

In addition, the study showed that the patients' satisfaction level was significantly associated with the duration of the teleconsultations. Patients who had their teleconsultation between 15 and 20 min appeared to be significantly more satisfied than the rest of the patients. Hence, it would be recommended for guidelines to set this duration as ideal for telehealth consultation.

The telehealth experiences investigated in the current study were positively rated by the patients across all domains of health care quality: safety, patient-centeredness, effectiveness and efficiency, timeliness, and access and equity. These parameters were initially defined by the IoM (54–56) and in turn endorsed by the WHO (57) as the basis of any healthcare delivery system.

Accordingly, in the context of this study, telehealth consultation was deemed by the patients to be safe, where they valued the intentions underlying the care that they received and perceived the corresponding experiences to entail no harm. This was supported by both the quantitative and the qualitative analysis. This finding may be influenced by the fact that contracting a COVID-19 infection was the most pressing safety concern while conducting the study, especially before having the vaccine readily available in the UAE. Hale and Kvedar (58) expressed similar findings even before the onset of the COVID-19 pandemic.

Most patients did not have concerns regarding telehealth prior to the consultation. The patients who had concerns regarding the quality-of-care prior to consultation, though, were found to be significantly less satisfied than patients who did not have concerns prior to consultation. This finding is in conjunction with previously conducted studies that show that patients who assume that the quality of the telehealth services is lagging and/or are not given the option of choosing between telehealth or otherwise tend to be less satisfied than others (59, 60).

The services were considered patient-centered, and hence, responsive to individual patient preferences, needs, and values. This was evident in the patients' feedback, where most of them selected “personal preference” as a reason for resorting to telehealth. The output of the qualitative analysis also showed that the patients found it very convenient to virtually attend telehealth appointments rather than having to physically go themselves. They also reported on the perceived privacy integral to meeting online rather than in person. These findings were in accordance with previous studies that found that patients were more likely to choose telehealth appointments over traditional consultations due to its convenience (24, 61). In addition, due to the COVID-19 pandemic, patients felt it was safer to meet online rather than having to risk exposure to COVID-19 virus. Also, it has been widely recognized that patients appreciate the ability to access their medical notes and results from home which is integral to telehealth medicine (11). Yet, some of the challenges that the patients indicated included the fact that there was the absence of human touch. Czartoski (62) recognizes this deficiency and adds more on how difficult it is to appreciate the patients values and preferences without the physical presence and proximity to the patient. This calls for continuous improvement efforts to be directed toward developing the physicians' interpersonal skills (skilling, upskilling, and reskilling) and allocating sufficient time within the telehealth consultation to understanding the patients views and opinions and factoring them into decision-making (7). The patient's decision-making capacity should not be compromised with the use of telehealth services. Woo et al. (63) highlighted factors that increased the patients' involvement in decision-making when using telehealth. These factors included previous experience with using telehealth and knowledge of one's own medical condition and confidence with using the differing types of technologies. It might be worth incorporating for the development of teleconsultation skills to take part of the medical curricula to empower students and junior doctors for the increasing adoption of telehealth (64, 65).

Patients also mentioned that there were organization deficiencies including appointment formalities and billing services. Some patients faced technical issues while trying to connect to the telehealth consultation online. There were also issues with the high cost and insurance coverage. Finally, a lot of patients were concerned about the fact that the doctors cannot physically examine them or order further investigations. In some instances where the patients were formally asked to travel to the hospital to obtain imaging and to give blood samples, the video consultation appeared to them as futile and time-hindering.

With challenges comes room for improvements. The patients suggested developing the scheduling systems and enhancing the communications among healthcare providers. The difficulties faced by patients around payments call for revising prices and insurance policies (and in turn enacting evidence-driven decisions) when it comes to telehealth services. Furthermore, there are potential technological advancement that can be achieved. For instance, a method to measure vitals in the comfort of the patients' home. Simple enhancements around the quality of the image and sound of the consultation would lead to major improvements in the overall quality of the entailed experiences. These suggestions were in alignment with those identified by the patients in a similar study conducted in Makassar City, Indonesia (66).

The patients also determined the received telehealth consultation to be effective. The services appeared to be evidence-driven, to meet the respective services' preset objectives, and to reach those who need them, while avoiding underuse and misuse. Moreover, it was found that patients who received teleconsultation, as a follow-up appointment, were significantly more satisfied than others. Another previously conducted study in the United States of America (USA) found that patients perceived telehealth consultations to be more effective for follow up appointments. This study assessed the effectiveness of telehealth to substitute post-operative follow-up clinic visit, and the results showed that most of the patients accepted telehealth as a successful solitary means of follow-up with a high degree of satisfaction (67). Also, satisfaction rates with video calls were significantly higher compared to those with audio calls only. This finding is different than that generated from a study conducted in an Obstetrics and Gynecology clinic in the United States, where 99% of patients found that their needs were met with the audio calls only (68). Such a discrepancy may be because of the exceptionality of COVID-19 times. The physical distancing directives left human beings yearning for more interactivity (69), which could be why the video calls were considered more fulfilling for the patients, relative to audio calls. In fact, in the current study, some patients mentioned that they found a video-call consultation to be richer than a regular phone call as the physicians were more engaged and appeared to be more informative. This is congruent with a previously conducted research study, where patients with Parkinson's disease conveyed that video calls constituted a more effective, easier way to connect with expert neuroscience nurses (70).

Relevantly, the telehealth services were also described as efficient, where benefits of the available resources were maximized, and waste of equipment, supplies, ideas, and energy was avoided. Some patients highlighted that telehealth saved waiting and travel time. The current study also showed that patients who consulted for medication refill purposes were found to be significantly less satisfied than those who consulted for other reasons, which was opposite to what was initially anticipated. Originally, the assumption was that when patients already know their doctors and have a fair understanding of their clinical condition and corresponding medications, they would appreciate the ease of getting a medication refill from the comfort of their homes (21, 71). This discrepancy might be because the hospital group under investigation (at the time of the data collection) did not deliver the medication after the online consultation. The patients, or someone on their behalf, were still required to commute to the hospital to pick-up the prescribed medication. Fortunately, given the respective hospital group's continuous quest to excellence and patient-centeredness, home delivery service was launched (soon after completion of the current study's data collection) to refill prescriptions for registered patients with chronic conditions (72).

The patients also considered the received telehealth services to be timely, where waits (and sometimes harmful delays for both those who receive and those who give care) were avoided. Similarly, a randomized control trial conducted in a dermatological center highlighted the importance of focusing on shortening of the waiting time to increase patient satisfaction (73).

Most of the patients, in the current study, perceived their telehealth experience as efficacious, and some viewed this set-up to be even better, in meeting the consultation's preset targets, relative to the traditional options (i.e., face-to-face consultation or that over a regular phone call). Along these lines, previous studies suggested that the satisfaction with telehealth can be associated with equal or better clinical outcomes, and cost savings (74). Also, a systematic literature review showed that the patient satisfaction with telehealth is associated with several attributes including but not limited to improved outcomes of care, preferred modality, ease of use, cost saving, improved communication, and improved self-management (24).

It is also worth shedding light on studies that focused on a single discipline. For example, a pilot study which aimed at comparing patient satisfaction with prenatal genetic counseling performed via video conferencing vs. that performed on-site, showed a high level of patient satisfaction of video-conferencing relative to onsite consultation. The respective study suggested that telehealth can be utilized to offer this service to underserved populations (75). There is also another study that explored the advantages of telehealth to treat patients with Parkinson's disease. The results showed high patient satisfaction, reduced travel burden, equal clinical outcomes, and improved health care utilization (74). Along these lines, the services in the current study were also judged to be accessible and equitable, where the provision of care and its quality were not dependent on personal characteristics such as gender, ethnicity, geographic location, and socioeconomic status. Gurney et al. (76) suggested enabling a wider reach of patients to physicians of different level of expertise as one of the key advantages of telehealth medicine. So, it is widely recognized that telehealth provides non-discriminatory services. However, certain communities might face disparity based on socioeconomic status especially if insurance coverage or technology access is limited. Schwamm et al. (11) emphasizes that major barriers of telehealth equitability are factors like low socioeconomic status or low literacy. These might escalate health discrimination among patients who have limited access to technology or literacy. It would be key for policymakers to proactively address these barriers if they are to effectively institutionalize telehealth.

On a relevant note, patients in another study expressed appreciation of the accessibility of telehealth but were concerned about the completeness of the consultation and the accuracy of physical exam findings (77). Some of the participants of the current study expressed similar concerns. In another study, patients expressed concern toward telehealth and how it will create a new burden to healthcare workers which could subsequently affect their overall health and wellbeing (78).

One of the contributions of this study is the introduction of a tool developed for this study, which was proven to be internally reliable and externally valid in the context of this study. Up to the best of the authors' knowledge, there was no validated tool to measure patients' satisfaction with telehealth that is contextualized to the MENA region and that matches the intricacies of an emergency (such as COVID-19) (79). The constructed tool used non-medical vocabulary and was assembled in both English and Arabic languages. Together, they constitute the top languages used throughout the UAE, and hence, ensured the survey questions were comprehensible to the participants. The questionnaire was self- administered, thus eliminating the possibility of interviewer bias (80). There are several valuable tools that are developed and deployed elsewhere for a specific group of patients. For example, a previously conducted study in Arkansas among obstetric patients who received telehealth services in 2016 relied on a tailor-made internally validated tool (81). Another study refers to a validated tool that was utilized in Canada among patients with dementia (82). Additionally, the tool introduced in this study was designed to inquire for both quantitative and qualitative data. The tool was also translated into Arabic, which can facilitate its adoption in other Arabic-speaking nations and in non-Arabic speaking nations for native Arabic speakers.

Everyone is aspiring for the COVID-19 pandemic to reach an end soon. The findings of the current study further endorse the suggestions of continuing to provide telehealth services, and for it to be incorporated within all healthcare facilities especially the ones providing medical care to chronically ill or immunocompromised patients (83). Yet, since the main means of providing telehealth services is wireless communicational systems, the patient confidentiality becomes at stake. It has been repetitively mentioned that telemedicine consultations, compared to traditional visits, are more susceptible to breach in privacy and security which could be a barrier to their implementation (21). Hence, it is of utmost importance to instill all the necessary measure to ensure cyber security. Despite the awareness of this risk, some patients continue to choose telehealth services compared instead of traditional face-to-face consultations because they found the benefits to outweigh the risks (84).

While our results provide compelling information, where patients were clearly satisfied with their telehealth experiences, it is important to shed light on the study's limitations. This research work was conducted during the times of COVID-19, which constitutes one of its strengths. Yet, the exceptionality of this period limits the generalizability of the corresponding findings. As evident in this study, a substantial amount of the sensed fulfillment among patients was due to the reduced risk of getting infected and/or further contributing to the spread of the disease. Also, due to the physical distancing directives, patients, for most of the time, were advised to consult from home, and at some point, were totally barred from visiting the clinic in person (and hence, had no other choice but to use telehealth services). For future studies, it would be helpful to deploy the tool introduced in this study to capture the perception of the patients as the pandemic is (hopefully) subsiding. Moreover, having a sample size of 94 patients is not considered “small” (in absolute terms) for a mixed methods study design which is meant to offer thorough, systemic exploration and in-depth insight into lived experiences (39, 85, 86). Yet, in terms of generalizability (again), it would be interesting to conduct an investigative/deductive study of a larger (more representative) sample size. We trust that by assimilating the findings derived from such a research study with that generated from this largely inductive work would bring plenty of value in terms of reinforcing decision-making governing telehealth medicine in the UAE. This follow-up study could be longitudinal in nature to circumvent another limitation inherent to this study where the causality around the identified associations could not be established. It would be great for this study to capture the extent of satisfaction of the healthcare providers along with that of the patients, as previously suggested by other studies (87). Finally, this study was conducted in a single private hospitals group in Dubai, UAE. This could have limited the variability of the target population/study sample. As a future direction, it would be better to include a random selection of public and private healthcare delivery systems for the sample of the study to be more representative of the population of patients in the UAE.

## Conclusion

Telehealth has significantly evolved and has been playing an increasingly important role in healthcare, especially during the COVID-19 pandemic. The results of this study will inform decision-makers about the patients' perception of telehealth to maximize their readiness, and better prepare the healthcare sector for the potential resurgence of COVID-19 and/or the occurrence of any such crises.

## Data Availability Statement

The raw data supporting the conclusions of this article will be made available by the authors, without undue reservation.

## Ethics Statement

The study's ethical approval was granted by the Mohammed Bin Rashid University of Medicine and Health Sciences (MBRU), Institutional Review Board (Reference # MBRU-IRB-2020-028). The patients/participants provided their written informed consent to participate in this study.

## Author Contributions

All authors listed have made a substantial, direct, and intellectual contribution to the work and approved it for publication.

## Funding

This study was supported in part by the MBRU publication fund.

## Conflict of Interest

The authors declare that the research was conducted in the absence of any commercial or financial relationships that could be construed as a potential conflict of interest.

## Publisher's Note

All claims expressed in this article are solely those of the authors and do not necessarily represent those of their affiliated organizations, or those of the publisher, the editors and the reviewers. Any product that may be evaluated in this article, or claim that may be made by its manufacturer, is not guaranteed or endorsed by the publisher.
