# Supplementary material for: Telehealth to the Rescue During COVID-19: A Convergent Mixed Methods Study Investigating Patients' Perception
Source: Front Public Health. 2021 Nov 30;9:730647. doi: 10.3389/fpubh.2021.730647 (PMC8669510; doi:10.3389/fpubh.2021.730647)
Supplement: Supplementary file 1 [file Data_Sheet_1.PDF]

Appendix 1: Telehealth Consultation- Patient Satisfaction Survey

Segment 1. **Sociodemographic Information**

a) Gender
- ❑ Female
- ❑ Male

b) Age
- ❑ <18
- ❑ 18-35
- ❑ 36-55
- ❑ >55

c) Highest level of education completed
- ❑ No School
- ❑ Elementary
- ❑ Junior
- ❑ High School
- ❑ University (Undergraduate studies)
- ❑ Higher Education (i.e., Masters, MD, or PhD)

d) Nationality

Segment 2. **<u>Telehealth Consultation Overview</u>**

a) Was your most recent Telehealth Consultation with Mediclinic Middle East covered by your health insurance?

❑ Yes

❑ No

b) Which Mediclinic Middle East facility did you most recently Telehealth Consultation with:

c) What type of Telehealth Consultation did you use?

❑ Audio call (i.e., phone call)

❑ Video call

d) What was your reason for choosing to use Telehealth Consultation (as opposed to face-to-face consultation)? (Select all that apply)

❑ Had no other choice because outpatient visits were cancelled or deferred

❑ COVID 19: personal preference for social distancing

❑ Satisfied with previous Telehealth Consultation visit

❑ Curiosity/wanted to see how it works

❑ Difficulty to travel/ commute to the hospital

❑ At risk population (e.g., elderly, immunocompromised)

❑Recommended by a physician or friend

e) What was the purpose of your most recent Telehealth Consultation? (Select all that apply)

❑ New consultation

❑ Follow-up

❑ Test results review

❑ Medication refill

f) Which specialty did you have your most recent Telehealth Consultation with?

g) Did you have any concerns <u>prior to</u> trying Telehealth Consultation at Mediclinic Middle East? (Select all that apply)

❑ No, I did not have any concerns

❑ Yes, I was worried about my confidentiality

❑ Yes, I was worried that the virtual set-up will prevent the physician from fully understanding my condition

❑ Yes, I was worried about technical difficulties (i.e., image and video quality, and audio issues)

❑ Yes, I was worried it will not as effective as in-person visits

❑Yes, I was worried it will not be covered by my insurance

❑Yes, I was concerned of the quality of the service since the physician will not be able to physically examine me

h) Relative to reaching out to your physician directly (on their mobile devices or so), how satisfied were you with your most recent Telehealth Consultation?

❑ Very Satisfied

❑ Satisfied

❑ Neutral

❑ Dissatisfied

❑ Very Dissatisfied

i) Relative to regular, face-to-face consultation (i.e., in person hospital visit), how satisfied were you with your most recent Telehealth Consultation?

❑ Very Satisfied

❑ Satisfied

❑ Neutral

❑ Dissatisfied

❑ Very Dissatisfied

j) Was there anything about using Telehealth Consultation that you found confusing and/ or complicated?

❑ Yes

❑ No

k) Approximately, how long was the most recent Telehealth Consultation?

❑ <5 min

❑ 5-10 min

❑ >10 and <15 min

❑ 15-20 min

❑ >30 min

Segment 3. **<u>Quality of Telehealth Consultation</u>**

Using the scale below, please rate your level of satisfaction with each of the following aspects of your experience with Telehealth Consultation:

5 = Very satisfied
4 = Satisfied
3 = Neutral
2 = Dissatisfied
1 = Very dissatisfied
0 = Not applicable

		Rating
i.	Access to Telehealth Consultation	
ii.	Availability of preferred physician	
iii.	Ease of booking an appointment prior to the consultation	
iv.	The preparatory support that you got, from Mediclinic Middle East, prior to the consultation	
v.	Waiting time for the consultation	
vi.	Ease of remotely seeing the physician during consultation	
vii.	Ease of remotely hearing the physician during consultation	
viii.	Ease of seeing any images on the monitor during the consultation	
ix.	Ease of engaging with the physician during consultation	
x.	Communication with the clinician during consultation	
xi.	The extent to which the physician addressed your questions and concerns	
xii.	The treatment plan and patient educational materials you received	
xiii.	The physician’s performance/ ability to identify and address your health problem	
xiv.	The overall quality of care received during the consultation	

Segment 4. **<u>Sustainability and Future Utilization</u>**

a) **How likely are you to resort to Telehealth Consultation at Mediclinic Middle East again?**
❏ Very likely
❏ Likely
❏ Neutral
❏ Unlikely
❏ Very unlikely

b) **How likely are you to recommend Telehealth Consultation at any of the units of Mediclinic Middle East in Dubai to a friend or a family member?**
❏ Very likely
❏ Likely
❏ Neutral
❏ Unlikely
❏ Very unlikely

c) **How likely do you think Telehealth Consultation will be used in the future as a primary means of consultation?**
❏ Very likely
❏ Likely
❏ Neutral
❏ Unlikely
❏ Very unlikely

d) **How satisfied would you be if Telehealth Consultation became the primary means of consultation in the near future?**
❏ Very Satisfied
❏ Satisfied
❏ Neutral
❏ Dissatisfied
❏ Very Dissatisfied

e) **What were the strengths of your Telehealth Consultation experience?**

f) **What were the weaknesses of your Telehealth Consultation experience?**

g) **What do you think could be done to improve the Telehealth Consultation that you recently experienced?**
